# Infected Peri-Pancreatic Necrosis Causing Gallbladder Necrosis by Direct Extension

**DOI:** 10.1155/1993/14751

**Published:** 1993

**Authors:** Moshe Schein, Ahmad Assalia, Pavel Schmulevski, Vladimir Meislin, Moshe Hashmonai

**Affiliations:** Department of Surgery B Rambam Medical Center the Faculty of Medicine the Technion Institute of Technology, P.O. Box 9602 Haifa 31096 Israel

## Abstract

Acute acalculous cholecystitis may develop in patients suffering from necrotizing pancreatitis. Conversely, acute pancreatitis may complicate acute gallbladder disease. We present a case that lends support to the existence of another possibility: gallbladder necrosis caused by direct extension of the necrotizing pancreatitic process.


*HPB Surgery*, 1993, Vol. 7, pp. 77–79
Reprints available directly from the publisher
Photocopying permitted by license only

© 1993 Harwood Academic Publishers GmbH
Printed in the United States of America

### CASE REPORT

### INFECTED PERI-PANCREATIC NECROSIS CAUSING GALLBLADDER NECROSIS BY DIRECT EXTENSION

MOSHE SCHEIN, AHMAD ASSALIA, PAVEL SCHMULEVSKI, VLADIMIR MEISLIN and MOSHE HASHMONAI

*Department of Surgery B, Rambam Medical Center, P.O. Box 9602, Haifa 31096, and the Faculty of Medicine, the Technion Institute of Technology, Israel*

*(Received 1 August 1992)*

Acute acalculous cholecystitis may develop in patients suffering from necrotizing pancreatitis. Conversely, acute pancreatitis may complicate acute gallbladder disease. We present a case that lends support to the existence of another possibility: gallbladder necrosis caused by direct extension of the necrotizing pancreatitic process.

KEY WORDS: Acalculous cholecystitis, acute pancreatitis, infected pancreatic necrosis

### INTRODUCTION

Acute acalculous cholecystitis is well known to be associated with critical diseases and to develop in patients suffering from burns, trauma, receiving parenteral nutrition, following major operations or any debilitating state[1]. Necrotizing pancreatitis and its infected complications constitute a severe disease which require extensive surgical treatment[2]. Obviously, acalculous cholecystitis can also develop in acute pancreatitis patients. Conversely, acute pancreatitis is known to occur following any surgical procedure or critical disease[3]. In fact, the mechanisms which initiate the necrotizing process in both acute pancreatitis and acalculous cholecystitis could be identical[4]. Consequently, the association between the two entities, acalculous cholecystitis developing in patients suffering from acute pancreatitis, or the latter complicating the former, has been reported sporadically[1,5].

The other possibility, that necrosis of the gallbladder may occur due to direct extension of the necrotizing peri-pancreatic process, is not mentioned in the literature. The following case supports our contention that this may indeed occur.

### CASE REPORT

A 62 years old male patient was admitted with severe acute alcoholic pancreatitis (APACHE II -17). Contrast enhanced CT scan was performed during the second week of the disease and disclosed patchy pancreatic uptake and a large subhepatic

Address correspondence to: M. Schein collection. Percutaneus drainage revealed necrotis material and pus. Concomitantly the patient developed massive upper gastrointestinal haemmorhage; endoscopy revealed chronic duodenal ulcer with an adherent clot. Haemorrhage persisted and laparotomy was undertaken. At operation the bleeding ulcer was underrun and truncal vagotomy/pyloroplasty performed. The pancreas appeared hard and viable but there was an extensive process of infected peripancreatic necrosis occupying both gutters and extending into the duodenohepatic ligament, up the porta hepatis. The gallbladder was adherent to the involved duodenohepatic ligament; it was necrotic at this point and perforated with a free subheptic collection of bile. Because of the obliteration in Calot's triangle partial cholecystectomy was performed[6]. Brisk bleeding from the edges of the resected gallbladder attested to the patency of the cystic artery. The necrotic process was debrided and drained. Postoperatively the abdominal infection persisted, complicated by leakage from the closure of the pyloroplasty, and multi-organ failure. Subsequently the abdominal cavity was managed with "laparostomy" and 12 planned re-laparotomies were necessary to reverse the septic process. The duodenal fistula closed spontaneously and he was discharged after 7 weeks of hospitalization. Examination of the gallbadder specimen disclosed an oedematous and thickened wall with areas of necrosis and perforation. There were yellow spots of fat necrosis. Histological examination revealed acute, necrotizing cholecystitis. There was evidence of fat necrosis and calcification in the outer layers of the wall.

### DISCUSSION

Numerous publications have documented that the necrotizing pancreatic process may involve adjacent visceral structures. Colonic necrosis is the most frequently described example[7] but small-bowel[8] and gastric[9] involvement are less well recognized conditions. Miller *et al.* have reported a patient in whom pancreatic necrosis eroded the intrapancreatic portion of the common bile duct[10]. If most juxtapancreatic viscera can be affected by the pancreatitic necrotizing process why not the gallbladder?Wwe believe that this, in fact, happened in our patient.

Various theories have been proposed to explain the associated necrosis of juxtapancreatic organs[7]. Firstly, it may be involved by direct contact with a highly septic exudate, rich in vasoactive substances and enzymes. Lending support to this hypothesis is the histologic observation (shown also in the present case) that the external layers of the viscus are more affected than the inner layers, as necrosis spreads "from outside to the inside". Secondly, thrombosis of supplying arteries or veins is another possible mechanism. Finally, shock and the occurrence of a low-flow state may account for ischaemia and subsequent gangrene.

Previously we have hypothesized that acute pancreatitis may adversely affect the healing of adjacent intestinal anastomoses[3]. The fact that the present case developed a leak from a pyloroplasty suture line, bathed in necrotizing pancreatic secretions, lends credibility to that view.

We conclude that the necrotizing pancreatic process may involve, by direct extension, the nearby gallbladder as well as any adjacent organ.

### References

1. Glenn, F. (1979) Acute acalculous cholecystitis. *Ann.Surg.*, **189**, 458–465
2. D'Egidio, A. and Schein, M. (1991) Surgical strategies in the treatment of pancreatic necrosis and infection. *Br.J.Surg.*, **78**, 133–137
3. Schein, M., Saadia, R. and Decker, G.A.G. (1988) Postoperative pancreatitis — a cause for anastomotic leaks. *S.Afr.Med.J.*, **73**, 550–551
4. Aldrige, M.C., Francis, N.D., Glazer, G. and Dudley, H.A.F. (1986) Colonic complications of severe acute pancreatitis. *Br.J.Surg.*, **76**, 362–367
5. Gemsenjager, E. (1991) Variable course in acute pancratitis exemplified by case reports (In German). *Schweiz.Rundsch.Med.Prax.*, **80**, 739–745
6. Schein, M. (1991) Partial cholecystectomy in the emergency treatment of acute cholecystitis in the compromised patient. *J.R.Coll.Surg.Edinb.*, **36**, 295–297
7. Schein, M., Saadia, R. and Decker, G.A.G. (1985) Colonic necrosis in acute pancreatitis. *Dis.Colon.Rectum*, **28**, 948–950
8. Doberneck, R.C. (1989) Intestinal fistula complicating necrotizing pancreatitis. *Am.J.Surg.*, **158**, 581–584
9. Warshaw, A.L., Moncure, A.C. and Rattner, D.W. (1989) Gastrocutaneous fistulas associated with pancreatic abscesses. *Ann.Surg.*, **210**, 603–607
10. Miller, B.M., Traverso, W. and Freeny, P.C. (1988) Intrapancreatic communication of bile and pancreatic ducts secondly to pancreatic necrosis. **123**, 1000-1003

*(Accepted by S. Bengmark 21 January 1993)*

### INVITED COMMENTARY

The authors describe an apparently unique entity namely the occurrence of perforation of the gallbladder in the presence of necrotizing pancreatitis. Whether this is in fact a progression of the necrotizing process which affects the pancreas or is in fact an example of perforation of the gallbladder in the presence of acalculous cholecystitis is unclear. Clearly the presence of bleeding from the cut edge of the gall bladder eliminates the possibility of cystic artery thrombosis with infarction of the gall bladder. That the necrotizing process affects the colon and duodenum with fistula formation is not a valid analogy since more often than not, perforation into the colon and duodenum occurs in association with therapy (multiple laparotomies) which in the face of intense inflammation may result in injury to viscera. Moreover, most fistula are of a more chronic nature rather than the acute process described in the present communication. Nevertheless, we are grateful to the authors for drawing our attention to the coexistence of gall bladder perforation in the face of pancreatic necrosis.

Raymond Doberneck
Dept of Surgery
Univ. of New Mexico
Albuquerque, USA

